# Effect of Ellagic Acid on Seizure Threshold in Two Acute Seizure Tests in Mice

**DOI:** 10.3390/molecules26164841

**Published:** 2021-08-10

**Authors:** Mateusz Pieróg, Katarzyna Socała, Elżbieta Wyska, Ewa Poleszak, Piotr Wlaź

**Affiliations:** 1 Department of Animal Physiology and Pharmacology, Institute of Biological Sciences, Maria Curie-Sklodowska University, Akademicka 19, PL 20-033 Lublin, Poland; katarzyna.socala@mail.umcs.pl; 2 Department of Pharmacokinetics and Physical Pharmacy, Jagiellonian University Medical College, Medyczna 9, PL 30-688 Kraków, Poland; e.wyska@uj.edu.pl; 3 Laboratory of Preclinical Testing, Chair and Department of Applied and Social Pharmacy, Medical University of Lublin, Chodźki 1, PL 20-093 Lublin, Poland; ewa.poleszak@umlub.pl

**Keywords:** ellagic acid, mice, seizure models, pentylenetetrazole, seizure threshold

## Abstract

Ellagic acid (EA) is a natural dietary polyphenol that has many beneficial properties, including anti-inflammatory, antioxidant, antiviral, antibacterial, and neuroprotective effects. Studies have revealed that EA may modulate seizure activity in chemically induced animal models of seizures. Therefore, the aim of the present study was to investigate the effect of EA on the seizure threshold in two acute seizure tests in male mice, i.e., in the intravenous (i.v.) pentylenetetrazole (PTZ) seizure test and in the maximal electroshock seizure threshold (MEST) test. The obtained results showed that EA (100 mg/kg) significantly elevated the threshold for both the first myoclonic twitch and generalized clonic seizure in the i.v. PTZ seizure test. At the highest dose tested (200 mg/kg), EA increased the threshold for tonic hindlimb extension in the MEST test. EA did not produce any significant changes in motor coordination (assessed in the chimney test) or muscular strength (investigated in the grip-strength test). The plasma and total brain concentration-time profiles of EA after intraperitoneal and oral administration were also determined. Although further studies are necessary to confirm the anticonvulsant activity of EA, our findings suggest that it may modulate seizure susceptibility in animal models.

## 1. Introduction

Epilepsy is a chronic neurological disease diagnosed in approximately 65 million people worldwide [1]. The pathomechanism of this disease is still not fully understood. Pharmacotherapy is the main form of treatment for epilepsy, but about 30% of epileptic patients remain drug-resistant [2]. Moreover, antiseizure drugs (ASDs) can cause numerous side effects when used alone or in polytherapy. Therefore, up to 25% of patients discontinue pharmacotherapy [3]. There is a need to find more effective and safer ASDs, and to explore new therapeutic strategies for epileptic disorders. Plants seem to be a promising source of compounds with anticonvulsant properties.

Ellagic acid (EA) is a dietary polyphenol found in many natural products. It is present, as a subfraction of ellagitannins, in many fruits, vegetables, herbs, and nuts [4,5]. Many studies have shown that EA impacts antioxidant and anti-inflammatory mediators, reduces lipid peroxidation, and scavenges free radicals in mammalian tissues and cells [6,7,8,9,10,11,12]. Moreover, EA exhibits antiviral [13], antibacterial [14], and antiproliferative [15] activities as well as providing chemoprotection against several carcinogens [16,17,18]. The intrinsic antioxidant capacity of EA is responsible for its broad-spectrum pharmacological properties, including the protection of neurons in neurodegenerative diseases [19,20,21]. Preclinical data from in vivo models demonstrated that EA ameliorates motor disturbances and neuro-inflammatory biomarker levels in a rat model of Parkinson’s disease [22]. Moreover, treatment with this polyphenol may represent a novel approach to alleviating the symptoms of multiple sclerosis [23], Alzheimer’s disease [24,25,26], or traumatic brain injury [27]. The potential antidepressant activity of EA was also proposed on the basis of its ability to control depressive-like behavior in rodents [28,29,30,31]. To date, information on the antiepileptic properties of EA is very limited. In one study by Dhingra and Jangra [32], acute (40 mg/kg) and chronic administration of EA (20 and 40 mg/kg) significantly reversed the pentylenetetrazole- (PTZ 80 mg/kg, administrated intraperitoneally, i.p.) and picrotoxin-induced (6 mg/kg, i.p.) convulsions in mice. It is worth noting that EA is poorly absorbed and is further metabolized by the gut microbiota [33,34,35,36], which may minimize the therapeutic benefit and limit the clinical use of EA after oral (p.o.) administration. To overcome this challenge, some delivery systems and preparations have been developed to enhance its bioavailability [37]. Interestingly, El-Missiry et al. [38] demonstrated that EA coated in calcium-alginate nanoparticles (Ca^2+^-EA-ALG NPs) exhibits higher anticonvulsant and neuroprotective potential than unencapsulated EA (50 mg/kg) against PTZ-induced brain impairment and convulsions in mice.

The data from pharmacokinetic studies [23,35,36] reveal that, when ingested, most EA is metabolized by the microbiota into dibenzopyran-6-one derivatives—urolithins (UROs). These metabolites are better absorbed and reach tissues more easily to potentially exert their beneficial effects, e.g., after blood-brain barrier (BBB) penetration; however, they have poor antioxidant capacity compared to EA [39]. According to Gasperotti et al. [40], UROs can be detected both in the plasma and in the brain of rats administered intravenously (i.v.) with EA. On the other hand, the results of the study by Yan et al. [41] proved that EA (50 mg/kg, p.o.) was detectable in several rat tissues, including the plasma, kidney, liver, heart, lung, and brain. Regarding the neurotherapeutic properties of EA, it seems that the effects of in vivo testing require confirmation of EA’s ability to cross the BBB, in order to justify treatment using it.

The present study was undertaken to evaluate the effect of EA on seizure thresholds in two acute seizure tests in mice: an i.v. PTZ seizure test and a maximal electroshock seizure threshold (MEST) test. Moreover, the acute adverse effects of EA on neuromuscular strength (in the grip-strength test) and motor coordination (in the chimney test) were investigated. The plasma and total brain concentration-time profiles of EA after i.p. and p.o. administration were also determined.

## 2. Results

### 2.1. Brain and Serum Concentrations of EA in Mice

**Figure 1:**
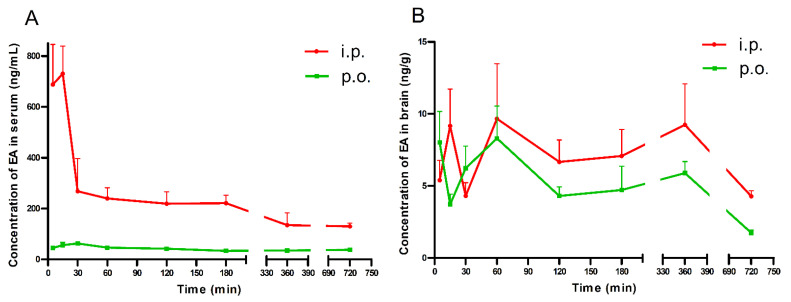
Serum (**A**) and brain (**B**) concentrations of EA in mice. Data are presented as the mean + standard error of the mean (SEM). EA (100 mg/kg) was administrated i.p. or p.o. 5, 15, 30, 60, 120, 180, 360, and 720 min before the mice were decapitated. Experimental groups consisted of 3–5 animals.

Brain and serum concentrations of EA after its acute i.p. and p.o administration (100 mg/kg) are shown in Figure 1A,B.

After the p.o. administration, the highest serum concentration of EA (62.43 ng/mL) was noted 30 min after treatment. Furthermore, EA reached the peak serum concentration (730.44 ng/mL) at 15 min after i.p. injection. Similarly, the mean concentrations in serum were over 4 and 5 times higher at 30 and 60 min after i.p. injection, respectively, than after p.o. administration (Figure 1A).

After p.o. and i.p. administration, the highest brain concentration of EA was noted 60 min after treatment (8.30 and 9.67 ng/g, respectively (Figure 1B)).

### 2.2. Effect of EA in the PTZ-Induced Seizure Threshold Test

**Figure 2:**
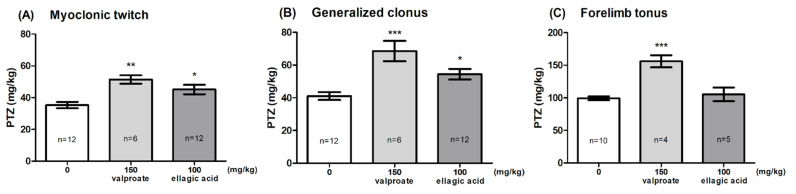
Effect of ellagic acid on the threshold for the onset of the first myoclonic twitch (**A**), generalized clonus (**B**), and forelimb tonus (**C**) in the i.v. PTZ seizure threshold test in mice. Ellagic acid and valproate (positive control) were injected i.p. 60 and 15 min before the test, respectively. The doses are shown on the abscissa. Control animals received 0.5% methylcellulose. Data are expressed as means (mg/kg PTZ) ± SEM. * *p* < 0.05, ** *p* < 0.01, *** *p* < 0.001 vs. the control group (one-way ANOVA followed by Dunnett’s post hoc test).

The effect of EA on the seizure threshold in the i.v. PTZ test is shown in Figure 2A–C (one-way ANOVA: *F* (2,27) = 8.347, *p* < 0.0015 for myoclonic twitch; *F* (2,27) = 12.78, *p* < 0.0001 for generalized tonus; *F* (2,16) = 19.06, *p* < 0.0001 for forelimb tonus). EA, administered at the dose of 100 mg/kg, significantly affected the susceptibility of mice to the PTZ-induced first myoclonic twitch and generalized clonic seizures with the loss of the righting reflex (Dunnett’s post hoc test: *p* < 0.05 vs. control group); however, it did not affect the threshold for forelimb tonus. VPA (150 mg/kg) significantly increased the threshold for the onset of all the studied endpoints (Dunnett’s post hoc test: *p* < 0.01 for myoclonic twitch; *p* < 0.001 for generalized clonus and forelimb tonus).

### 2.3. Effect of EA in the MEST Test

**Figure 3:**
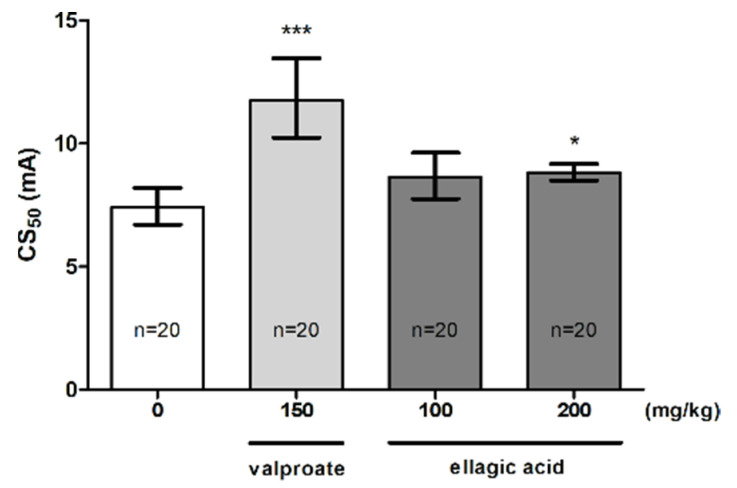
Effect of ellagic acid on the seizure threshold in the MEST test in mice. Ellagic acid and valproate (positive control) were injected i.p. at 60 and 15 min before the test, respectively. The doses are shown on the abscissa. Control animals received 0.5% methylcellulose. Data are expressed as CS_50_ (in mA) values with 95% confidence limits. Each CS_50_ value represents the current intensity predicted to produce convulsions in 50% of mice. * *p* < 0.05, *** *p* < 0.001 vs. the control group (one-way ANOVA followed by Dunnett’s post hoc test).

The influence of EA on the threshold for the tonic hindlimb extension in the MEST test is shown in Figure 3 (one-way ANOVA: F (3,33) = 14.40, *p* < 0.0001). EA at a dose of 100 mg/kg did not affect the seizure threshold. However, a statistically significant increase in the CS_50_ value was observed for a dose of 200 mg/kg (*p* < 0.05). Positive control (VPA at 150 mg/kg) significantly increased the seizure threshold (*p* < 0.001 vs. the vehicle-treated group).

### 2.4. Effects of EA on Motor Coordination and Muscular Strength in Mice

Table 1 presents the influence of EA at doses of 100 and 200 mg/kg on neuromuscular strength and motor coordination in mice. There were no significant impairments of motor coordination in the chimney test (Fisher’s exact test: *p* > 0.05). Moreover, EA at any of the doses tested had no effect on muscular strength, as assessed by the grip strength test (one-way ANOVA: *F* (3,36) = 1.088, *p* = 0.367).

## 3. Discussion

Due to its multi-faceted action, EA has many potential health benefits for humans [20]. Among these mechanisms of action, free radical scavenging, iron chelation, the activation of different cell signaling pathways, and the mitigation of mitochondrial dysfunction are often mentioned. Its protective effects against several neurodegenerative disorders have also been highlighted [19]. Moreover, recent reports suggest that EA may demonstrate anticonvulsant activity [32,38]. Here, we provide novel data on the effect of EA in the control of seizure thresholds in the i.v. PTZ and MEST test.

The absorption of EA in animals is very poor, and EA usually undergoes extensive metabolism by the gut microbiota to produce UROs [42]. Therefore, evaluation of the pharmacokinetic and tissue distribution profiles of EA is important. Yan et al. [41] showed that after oral administration of EA at 50 mg/kg, EA was detected in many rat tissues. Notably, EA was detected in the brain at a relatively low concentration at 0.5, 1, 2 and 4 h after treatment. In order to characterize the ability of EA to cross the BBB after the p.o. and i.p. route of administration in mice, we performed a pilot pharmacokinetic study. We have shown that changes in the serum concentrations of EA (100 mg/kg) following i.p. and p.o. administration follow a similar time-course pattern. The maximum concentrations were reached at almost the same time points, i.e., at about 30 min after p.o. administration and 15 min after i.p. injection. Some reports suggested that EA has poor absorption, rapid distribution and elimination, which may prevent animal tissues from attaining and maintaining sufficiently high concentrations [43,44,45]. Oral administration appears to be the most preferred route of administration of EA; however, there are many factors that can complicate its absorption. EA is a weak acid, which is ionized at the physiological pH (4); it also forms poorly soluble complexes with divalent ions, such as Mg^2+^ and Ca^2+^, and eventually, EA is metabolized by intestinal microorganisms [42,44,46]. All of the above can inhibit the biological activity of this polyphenol. In studies by Teel and Martin [45] and Lei et al. [43], the maximal blood level of EA in rodents was reached 30 min after the p.o. administration of its synthetic form or as extract of pomegranate leaf (*Punica granatum*, a natural source of EA). Furthermore, Smart et al. [44] and Teel [47] showed that the concentration of EA in mice blood was highest within the first 15–30 min after i.v. or i.p. administration, and it decreased rapidly over time. The present study is in line with the abovementioned reports. In one study by Teel [47], the amount of EA in the mouse brains was low and was approximately constant over the time period from 15 min to 24 h. The authors indicated that this likely reflects the relative impermeability of the BBB to EA. In our study, the maximum EA concentrations in the mouse brains correspond with those described by Yan and colleagues [41], and were reached at about 60 min after p.o. and i.p. administration. In the study by Farbood et al. [27], in rats pretreated with EA (p.o.) at a dose of 100 mg/kg for 7 consecutive days, the BBB function was normal; therefore, it can be concluded that EA did not cause severe BBB disturbances. It seems that, despite its low concentration in animal brains, EA may exert central effects. The pretreatment time of 60 min for EA was used in all subsequent experiments to study its acute effects on seizure thresholds.

In the i.v. PTZ seizure threshold test, EA (100 mg/kg) slightly increased the seizure threshold for the onset of both the first myoclonic twitch and the generalized clonic seizures. A potentially high dose of this polyphenol was unable to slow down the progression of seizures from generalized clonus to forelimb tonic extension. This test is considered as an extremely sensitive parametric method for assessing seizure thresholds in rodents [48]. The proconvulsant activity of PTZ is at least partially mediated by its ability to block the chloride ion channel in the γ-aminobutyric acid type A (GABA_A_) receptor complex, and the PTZ seizure thresholds are particularly sensitive to compounds that affect GABAergic neurotransmission [49]. Studies by Dhingra and Jangra [32] and El-Missiry et al. [38] suggest that free and encapsulated EA (Ca^2+^-EA-ALG NPs) at doses of 20–50 mg/kg inhibited PTZ- and/or picrotoxin-induced convulsions in mice through an increase in GABA levels in the brain. Thus, it seems that an increased seizure threshold for myoclonic twitch and generalized clonus following EA administration could have resulted from the enhancement of GABAergic neurotransmission.

In the next experiment, EA at a higher dose (200 mg/kg) significantly raised the seizure threshold for the tonic hindlimb extension in the MEST test. As a model of generalized tonic-clonic seizures in humans, the MEST test may be useful in detecting anticonvulsants that are acting as sodium channel blockers [50]. Therefore, it seems that EA at a higher dose also inhibits tonic seizures by blocking sodium channels. To date, there is no data on the direct influence of this compound on the inhibition of sodium influx through cell membranes.

There is clear evidence of the involvement of inflammatory processes in epileptogenesis [51]. Seizures are associated with increased mitochondrial membrane oxidative stress and the downregulation of glutathione homeostasis in the hippocampus [52,53,54]. Data suggest that high levels of proinflammatory cytokines are risk factors for epilepsy, and that anti-cytokine agents are therapeutic for epilepsy [55]. Some authors also suggest that combating oxidative stress is a key approach to improving disease outcomes in epilepsy [56]; thus, antioxidants may be an effective treatment. PTZ significantly increased the proinflammatory mediators in mice, such as IL-6 and TNF-α [38]. Furthermore, after PTZ-induced seizures, significant decreases in GSH, cysteine, glutathione disulfide, and protein thiols, as well as increases in the protein disulfides and protein carbonyl levels, were observed in the mouse hippocampus [57]. It is worth mentioning that EA has been reported to show antioxidant activity [58], with efficacy in reducing IL-6, IL-1β, and TNF-α levels, in inhibiting NF-κB action and also in normalizing lipid metabolism and the lipidemic profile [26,27,38,59,60].

Some preclinical studies indicate that the dose-related antinociceptive action of EA has both peripheral and central components that involve mediation by the opioidergic system and the l-arginine-NO-cGMP-ATP-sensitive K^+^ channels pathway [61,62,63]. In another study, Olgar et al. [64] showed that EA suppresses Ca^2+^ currents in rat ventricular myocytes and exerts negative inotropic effects through the activation of NOS-GC-cGMP pathways. It should be noted that NO is a multifunctional signaling molecule, but it is also a key mediator of excitotoxic neuronal damage and is involved in the neurobiology of many diseases, including epilepsy [65,66,67]. According to Prast and Philippu [68], the NO/cGMP pathway may be involved in GABA-mediated transmission and glutamate-mediated excitatory transmission. The release of GABA is biphasically dependent on the concentration of NO. Basal NO levels decrease GABA release, but high concentrations of NO enhance GABA outflow. Thus, it seems that an increased seizure threshold following EA administration could have resulted from a high NO concentration and a subsequent increase in GABA-mediated neuronal inhibition. The glutamate-mediated excitatory transmission process also seems to be controlled by NO/cGMP in a biphasic manner. In general, guanylyl cyclase is activated by NO, and it increases the cGMP level and suppresses glutamate release; however, very high cGMP concentrations increase the release of glutamate [68]. Hence, EA at the doses used in our study could have also inhibited the release of this excitatory neurotransmitter. We can only speculate that EA (as a NO/cGMP modulator) may modulate inhibitory and excitatory neurotransmission and/or some ion channel functioning. This issue needs further investigation. EA also has other in vivo effects, i.e., antidepressant- or anxiolytic-like effects, suggesting its ability to modulate the monoaminergic pathways (serotonergic, noradrenergic, dopaminergic) [28,69]. Clinical data has revealed that monoamines play an important role in regulating epileptogenesis, seizure susceptibility, and convulsions [70]. The antidepressant activity of EA may also be mediated by modulation of the endogenous production of brain-derived neurotrophic factor (BDNF) [30]. Studies have shown that BDNF is upregulated in areas implicated in epileptogenesis, and interference with BDNF signal transduction inhibits the development of the epileptic state [71]. Although further studies are needed to confirm which mechanisms are responsible for the anticonvulsant activity of EA, the results of in vitro and in vivo studies provide a rationale for considering this polyphenol as a compound with the potential to modulate seizure susceptibility in animal models.

Bhandary et al. [72] demonstrated that the pomegranate extracts, as well as the synthetic EA, at levels up to 2000 mg/kg body weight did not cause any adverse effects and may be considered nontoxic and safe. Furthermore, Patel et al. [73] showed that, in a study of the acute toxicity of standardized pomegranate extract in rats and mice, the oral LD_50_ of EA was greater than 5000 mg/kg body weight. It is worth mentioning that, in our study, none of the doses of EA produced any acute side effects as determined by the chimney test and the grip-strength test. 

In conclusion, the present study demonstrates that EA exerts slight anticonvulsant effects in two distinct seizure threshold tests in mice. Its antiseizure properties may result not only from the enhancement of GABAergic neurotransmission but also from the inhibition of sodium influx through cell membranes. It is possible that other mechanisms may be involved too. Further detailed studies of this multi-target bioactive compound in animal models of seizures and epilepsy are required. The possible interactions of EA with ASDs should be also assessed.

## 4. Materials and Methods

### 4.1. Animals

The study was carried out on adult male albino Swiss mice weighing 26–34 g. The animals were obtained from a licensed breeder (Laboratory Animals Breeding, Warsaw, Poland). Mice were housed in groups of 8–9 in Makrolon cages (37 cm × 21 cm × 14 cm) under controlled laboratory conditions (ambient temperature, 21–24 °C, relative humidity, 45–65%, 12 h light/dark cycle, light on at 7:00 a.m.). Chow pellets (Agropol S.J., Motycz, Poland) and tap water were available ad libitum. The mice were used after at least one week of acclimatization. All behavioral experiments were performed during the light phase between 8:00 a.m. and 3:00 p.m., after a minimum 30 min period of acclimatization to the experimental room. The animals were habituated to handling for one week prior to behavioral assays to minimize stress and its effects on experimental variability.

The total number of animals used in the present study was 190. The animals were assigned to the experimental groups as follows: 5 animals/group, in order to determine the pharmacokinetic study of EA, 6–12 animals/group in the intravenous PTZ test, 20 animals/group in the MEST test, and 10 animals/group in the grip-strength test and the chimney test. The grip-strength test and the chimney test are very quick and non-invasive procedures. Therefore, both tests were carried out on the same groups of animals shortly before the MEST test. This allowed us to limit the total number of animals used in the present study, which was in accordance with the guidelines of the Ethics Committee.

### 4.2. Drugs

EA (Sigma-Aldrich, St. Louis, MO, USA) was suspended in a 0.5% aqueous solution of methylcellulose (Sigma-Aldrich, St. Louis, MO, USA). Valproate (VPA, as sodium salt; Sigma-Aldrich, St. Louis, MO, USA) was dissolved in saline. EA was administered i.p. or p.o. by gastric gavage before the respective test. The gavage needle (curved) had a ball-shaped, smooth, rounded tip to prevent injury to the esophagus and other tissues. The needle was gently advanced along the upper palate until the esophagus of the mouse was reached. EA pretreatment time (60 min) in the seizure threshold tests was based on a pharmacokinetic study (see Figure 1A,B). VPA (positive control) was administered i.p. 15 min prior to the tests. All drug solutions/suspensions were administered at a volume of 10 mL/kg of body weight. The negative control groups received the vehicle only.

### 4.3. Pharmacokinetic Study of EA

EA was administered at the dose of 100 mg/kg (i.p. or p.o.) 5, 15, 30, 60, 120, 180, 360, and 720 min before the mice were decapitated. The collected blood samples (~1 mL/Eppendorf tube) were allowed to clot at room temperature. Blood samples were then centrifuged at 3000× *g* for 10 min and the serum was collected into new polyethylene tubes. Brains were removed from skulls and washed with 0.9% NaCl. The samples were kept at −25 °C until analysis.

High-performance liquid chromatography (HPLC) with an ultraviolet (UV) detection system was used to measure the serum levels of EA. First, 200 µL of serum samples were washed by shaking (IKA Vibrax VXR, Staufen, Germany) with 1 mL of ethyl acetate. The organic layers were discarded and the samples were spiked with 10 µL of internal standard (oxcarbazepine, 4 µL/mL). After vortex mixing (Reax top, Heidolph, Schwabach, Germany), 5 µL of 50% orthophosphoric acid was added to each sample and they were vortexed for 1 min. Then, 1 mL of methanol was added and the samples were shaken for 5 min. After centrifugation (Eppendorf miniSpin centrifuge), 650 µL of organic layers were transferred to new tubes and evaporated to dryness in a water bath (37 °C) under a gentle stream of nitrogen. The residues were dissolved in 100 μL of methanol and aliquots of 10 μL were injected into the HPLC system. The analysis was performed on a 250 × 4 mm LiChrospher^®^ 100 RP-18 column with a particle size of 5 μm (Merck, Darmstadt, Germany), protected with a guard column (4 × 4 mm) of the same packing material as the analytical column. The mobile phase was composed of 0.2% orthophosphoric acid water solution: methanol mixed at a ratio of 55:45 (*v*/*v*) and pumped at a flow rate of 1 mL/min. The column temperature was kept at 40 °C. The HPLC system (Merck-Hitachi, Darmstadt, Germany) consisted of an L-7100 isocratic pump, an L-7200 autosampler, and a UV variable-wavelength K-2600 detector (Knauer, Berlin, Germany) operating at 254 nm. Data acquisition and processing were performed using the D-7000 HSM software. In these conditions, the retention times of EA and internal standard were approximately 7 and 10 min. The calibration curve, constructed by plotting the ratios of the peak area of EA to internal standard versus EA concentrations, was linear in the tested concentration range, i.e., from 25 to 2000 ng/mL. No peaks were present in the chromatogram at the retention times of both compounds. The method was accurate and precise, with the accuracy expressed as a percentage of the nominal concentration, in the range of 94% to 106%, and the intra- and inter-day coefficients of variation (CV%) at less than 10%. The concentrations of EA in the mouse serum were expressed in ng/mL.

Because the concentrations of EA in brain tissue were below the limit of quantification via the HPLC/UV method, brain concentrations of this compound were measured by liquid chromatography tandem mass spectrometry (LC-MS/MS) using the Sciex QTRAP 4500 triple quadrupole mass spectrometer, coupled to the Excion LC AC HPLC system (both from Danaher Corporation, Washington, DC, USA). The brains were homogenized in distilled water (1:4, *w*/*v*) with a tissue homogenizer (TH220; Omni International, Inc., Warrenton, VA, USA). The homogenate samples (50 μL), after the addition of 5 μL of 50% acetic acid, were briefly vortexed and deproteinized at the ratio of 1:3, with 0.1% formic acid in acetonitrile containing an internal standard (valsartan). Then, they were shaken for 10 min (IKA Vibrax VXR, Staufen, Germany), and after centrifugation for 10 min at the speed of 8000× *g* (Eppendorf miniSpin centrifuge), the supernatant was transferred into the autosampler vials. Chromatographic separation was carried out on an XBridge™ C18 analytical column (3 × 100 mm, 5 µm, Waters, Ireland) with the oven temperature set at 30 °C. The autosampler temperature was maintained at 10 °C, and a sample volume of 10 μL was injected into the LC-MS/MS system. The mobile phase, containing 0.1% formic acid in acetonitrile (A) and 0.1% formic acid in water (B), was delivered at 0.4 mL/min using a gradient mode. The initial mobile phase composition was 95% B for the first 2 min, with a linear gradient to 20% B in the next 1 min, then in isocratic mode for 2 min, with a subsequent change back to 95% B in 1 min. The remaining time of elution was set at 95% B. The whole HPLC operation lasted 10 min. Electrospray ionization (ESI) in the negative ion mode was used for ion production. The tandem mass spectrometer was operated at unit resolution in the selected reaction monitoring mode (SRM), monitoring the transition of the deprotonated molecular ions *m/z* 301 to 284 (CE = −40 V) and *m/z* 301 to 229 (CE = −38 V) for EA (the first pair was used as a quantifier for quantification and the second as a qualifier for the identity verification), and *m/z* 434 to 179 for valsartan (IS). The mass spectrometric conditions were optimized for EA by a continuous infusion of the standard solution at the rate of 10 μL/min, using a Harvard infusion pump. The ion source temperature was maintained at 450 °C. The ion spray voltage was set at −4500 V. The curtain gas (CUR) was set at 40 psi, and the collision gas (CAD) at medium. Data acquisition and processing were accomplished using the Applied Biosystems Analyst software, version 1.7 (Sciex, Framingham, MA, USA). The calibration curves were constructed by plotting the ratios of the peak area of the analyte to IS versus analyte concentrations, and generated by a weighted (1/x × x) linear regression analysis. The quantitation ranges for this method were from 2.5 to 1000 ng/g of brain tissue, with accuracy from 92.85–115%. No significant matrix effect was observed, and there were no stability-related problems during the routine analysis of the samples. 

### 4.4. Intravenous PTZ Seizure Threshold Test

Following EA, VPA or vehicle administration, mice were placed in a cylindrical plastic restrainer (12 cm long, 3 cm inner diameter), and a 27-gauge needle (Sterican^®^, B. Braun Melsungen, Melsungen, Germany) was inserted into the lateral tail vein. The 2 cm long needle was attached by polyethylene tubing (PE20RW, Plastics One Inc., Roanoke, VA, USA) to a 10 mL plastic syringe containing 1% solution of PTZ (Sigma-Aldrich, St. Louis, MO, USA), which was mounted in a syringe pump (model Physio 22, Hugo Sachs Elektronik–190 Harvard Apparatus GmbH, March-Hugstetten, Germany). The correct placement of the needle in the tail vein was verified by the appearance of blood in the tubing. The needle was secured to the tail with adhesive tape. Following catheterization, the unrestrained mice were placed in a Plexiglas arena for behavioral observation. PTZ was administered to animals at a constant rate of 0.2 mL/min. During the test, mice were observed for the onset of different types of seizures by a trained and treatment-blind observer. The time that elapsed from the initiation of the PTZ infusion to the onset of three stages of seizures was measured, i.e., (1) the initial myoclonic twitch, (2) generalized clonus with loss of the righting reflex, and (3) tonic forelimb extension. The thresholds were calculated separately for each endpoint according to the formula: threshold dose of PTZ (mg/kg) = (infusion duration (s) × infusion rate (mL/s) × PTZ concentration (mg/mL) × 1000)/ body weight (g). Seizure thresholds were expressed as the amount of PTZ (mg/kg) ± SEM needed to produce the first sign of each endpoint in each experimental group. Tonic convulsions were usually lethal for mice. All surviving animals were euthanized immediately after the end of the infusion [74].

### 4.5. Maximal Electroshock Seizure Test

One minute before stimulation, a drop of ocular anesthetic (1% solution of tetracaine hydrochloride; Sigma-Aldrich, St. Louis, MO, USA) was applied to each eye of the mice. The maximal electroshock seizures were induced by constant current stimuli (50 Hz sinewave, 0.2 s) using a rodent shocker (type 221; Hugo Sachs Elektronik, Freiburg, Germany). An alternating sinusoidal current was administered through transcorneal electrodes, soaked in saline. During stimulation, mice were manually immobilized by the hand of the experimenter for 3–5 s. Immediately after stimulation, animals were placed in a Plexiglas arena for behavioral observation. The endpoint was a tonic extension of the hindlimb that exceeded a 90° angle with the body. The thresholds for maximal electroconvulsion were assessed by the “up-and-down” method described by Kimball et al. [75]. The current intensity was lowered or raised by 0.06-log intervals, depending on whether the previously stimulated animal did or did not exert tonic hindlimb extension, respectively [74]. Each mouse was stimulated only once at any given current intensity. The data obtained in groups of 20 animals were used to determine the threshold current-causing endpoint in 50% of the mice (CS_50_ with confidence limits for 95% probability).

### 4.6. Grip-Strength Test

The influence of EA on muscular strength in mice was determined via the grip-strength test (10 mice/group). The apparatus for this test (BioSeb, Chaville, France) consisted of a steel wire grid (8 × 8 cm) connected to an isometric force transducer. The mouse was lifted by its tail so that it could grasp the grid with its forepaws. The animal was then pulled back steadily by the tail until it released the grid. The maximal grip strength value (in newtons (N)) of the animal was recorded. The mean of three consecutive measurements for each animal was calculated. Body weight is a factor that affects the grip force; therefore, the mean force was normalized to body weight and expressed in mN/g ± SEM.

### 4.7. Chimney Test

The chimney test was used to detect the motor deficits in mice, as induced by EA. In this test, the inability of the animals (10 mice/group) to climb backward, up through a Plexiglass tube (3 cm, inner diameter × 30 cm, length), within 60 s was considered as a motor impairment [76]. Results were presented as the percentage of mice with motor coordination impairment.

### 4.8. Statistical Analysis

Data were analyzed by one-way analysis of variance (one-way ANOVA), followed by the Dunnett’s post hoc test, for multiple comparisons. In order to perform statistical analyses of data obtained in the MEST tests, the CS_50_ values with 95% confidence limits were transformed into the mean value of logarithms (of current strength) with standard deviation. Fisher’s exact probability test was used to compare the data from the chimney test. The statistical analysis was carried out using GraphPad Prism for Windows, version 5.03 (GraphPad Software, San Diego, CA, USA). *p <* 0.05 was considered to be statistically significant.

## Figures and Tables

**Table 1:** Effect of EA on skeletal muscular strength and motor coordination in mice.

Treatment (mg/kg)	Impairment of Motor Performance (%)	Neuromuscular Strength (mN/g)
Control	0	32.4 ± 1.2
VPA (150)	0	32.2 ± 0.8
EA (100)	0	32.3 ± 1.3
EA (200)	0	30.1 ± 0.9

Results are presented as a percentage of animals showing motor coordination impairment in the chimney test, and as a mean ± SEM of grip strength in millinewtons per gram of mouse body weight (mN/g) from the grip strength test, assessing skeletal muscular strength in mice, with *n* = 10 mice for each experimental group. The results from the grip-strength test were analyzed using a one-way ANOVA test. Fisher’s exact probability test was used to analyze the results from the chimney test.

## Data Availability

The data presented in this study are available on request from the corresponding authors.

## Sample Availability

Samples of the compounds are not available from the authors.

## References

[1] Moshe S.L., Perucca E., Ryvlin P., Tomson T. (2015). Epilepsy: New advances. Lancet.

[2] Billakota S., Devinsky O., Kim K.W. (2020). Why we urgently need improved epilepsy therapies for adult patients. Neuropharmacology.

[3] Perucca P., Gilliam F.G. (2012). Adverse effects of antiepileptic drugs. Lancet Neurol..

[4] Daniel E.M., Krupnick A.S., Heur Y.-H., Blinzler J.A., Nims R.W., Stoner G.D. (1989). Extraction, stability, and quantitation of ellagic acid in various fruits and nuts. J. Food Compos. Anal..

[5] Raudone L., Bobinaite R., Janulis V., Viskelis P., Trumbeckaite S. (2014). Effects of raspberry fruit extracts and ellagic acid on respiratory burst in murine macrophages. Food Funct..

[6] Zeb A. (2018). Ellagic acid in suppressing in vivo and in vitro oxidative stresses. Mol. Cell Biochem..

[7] Priyadarsini K.I., Khopde S.M., Kumar S.S., Mohan H. (2002). Free radical studies of ellagic acid, a natural phenolic antioxidant. J. Agric. Food Chem..

[8] Hwang J.M., Cho J.S., Kim T.H., Lee Y.I. (2010). Ellagic acid protects hepatocytes from damage by inhibiting mitochondrial production of reactive oxygen species. Biomed. Pharmacother..

[9] El-Garhy A.M., Abd El-Raouf O.M., El-Sayeh B.M., Fawzy H.M., Abdallah D.M. (2014). Ellagic acid antiinflammatory and antiapoptotic potential mediate renoprotection in cisplatin nephrotoxic rats. J. Biochem. Mol. Toxicol..

[10] Khanduja K.L., Avti P.K., Kumar S., Mittal N., Sohi K.K., Pathak C.M. (2006). Anti-apoptotic activity of caffeic acid, ellagic acid and ferulic acid in normal human peripheral blood mononuclear cells: A Bcl-2 independent mechanism. Biochim. Biophys. Acta.

[11] Saba, Khan S., Parvez S., Chaudhari B., Ahmad F., Anjum S., Raisuddin S. (2013). Ellagic acid attenuates bleomycin and cyclophosphamide-induced pulmonary toxicity in Wistar rats. Food Chem. Toxicol..

[12] Vieira O., Escargueil-Blanc I., Meilhac O., Basile J.P., Laranjinha J., Almeida L., Salvayre R., Negre-Salvayre A. (1998). Effect of dietary phenolic compounds on apoptosis of human cultured endothelial cells induced by oxidized LDL. Br. J. Pharmacol..

[13] Goodwin E.C., Atwood W.J., DiMaio D. (2009). High-Throughput Cell-Based Screen for Chemicals That Inhibit Infection by Simian Virus 40 and Human Polyomaviruses. J. Virol..

[14] Nohynek L.J., Alakomi H.L., Kahkonen M.P., Heinonen M., Helander I.M., Oksman-Caldentey K.M., Puupponen-Pimia R.H. (2006). Berry phenolics: Antimicrobial properties and mechanisms of action against severe human pathogens. Nutr. Cancer.

[15] Mishra S., Vinayak M. (2014). Ellagic acid induces novel and atypical PKC isoforms and promotes caspase-3 dependent apoptosis by blocking energy metabolism. Nutr. Cancer.

[16] Grossi M.R., Berni A., Pepe G., Filippi S., Meschini R., Papeschi C., Natarajan A.T., Palitti F. (2014). Evaluation of the effects of ellagic acid (EA) on 7,12-dimethylbenz(α) anthracene (DMBA) induced micronuclei in mammalian cells in vitro and in vivo. Toxicol. Lett..

[17] Zahin M., Ahmad I., Gupta R.C., Aqil F. (2014). Punicalagin and ellagic acid demonstrate antimutagenic activity and inhibition of benzo[a]pyrene induced DNA adducts. Biomed. Res. Int..

[18] Khanduja K.L., Gandhi R.K., Pathania V., Syal N. (1999). Prevention of *N*-nitrosodiethylamine-induced lung tumorigenesis by ellagic acid and quercetin in mice. Food Chem. Toxicol..

[19] Ahmed T., Setzer W.N., Nabavi S.F., Orhan I.E., Braidy N., Sobarzo-Sanchez E., Nabavi S.M. (2016). Insights Into Effects of Ellagic Acid on the Nervous System: A Mini Review. Curr. Pharm. Des..

[20] Alfei S., Turrini F., Catena S., Zunin P., Grilli M., Pittaluga A.M., Boggia R. (2019). Ellagic acid a multi-target bioactive compound for drug discovery in CNS? A narrative review. Eur. J. Med. Chem..

[21] Gupta A., Singh A.K., Kumar R., Jamieson S., Pandey A.K., Bishayee A. (2021). Neuroprotective Potential of Ellagic Acid: A Critical Review. Adv. Nutr..

[22] Sarkaki A., Farbood Y., Dolatshahi M., Mansouri S.M., Khodadadi A. (2016). Neuroprotective Effects of Ellagic Acid in a Rat Model of Parkinson’s Disease. Acta Med. Iran..

[23] Busto R., Serna J., Perianes-Cachero A., Quintana-Portillo R., Garcia-Seisdedos D., Canfran-Duque A., Paino C.L., Lerma M., Casado M.E., Martin-Hidalgo A. (2018). Ellagic acid protects from myelin-associated sphingolipid loss in experimental autoimmune encephalomyelitis. Biochim. Biophys. Acta Mol. Cell Biol. Lipids.

[24] Rojanathammanee L., Puig K.L., Combs C.K. (2013). Pomegranate polyphenols and extract inhibit nuclear factor of activated T-cell activity and microglial activation in vitro and in a transgenic mouse model of Alzheimer disease. J. Nutr..

[25] Hartman R.E., Shah A., Fagan A.M., Schwetye K.E., Parsadanian M., Schulman R.N., Finn M.B., Holtzman D.M. (2006). Pomegranate juice decreases amyloid load and improves behavior in a mouse model of Alzheimer’s disease. Neurobiol. Dis..

[26] Jha A.B., Panchal S.S., Shah A. (2018). Ellagic acid: Insights into its neuroprotective and cognitive enhancement effects in sporadic Alzheimer’s disease. Pharmacol. Biochem. Behav..

[27] Farbood Y., Sarkaki A., Dianat M., Khodadadi A., Haddad M.K., Mashhadizadeh S. (2015). Ellagic acid prevents cognitive and hippocampal long-term potentiation deficits and brain inflammation in rat with traumatic brain injury. Life Sci..

[28] Girish C., Raj V., Arya J., Balakrishnan S. (2012). Evidence for the involvement of the monoaminergic system, but not the opioid system in the antidepressant-like activity of ellagic acid in mice. Eur. J. Pharmacol..

[29] Ferreres F., Grosso C., Gil-Izquierdo A., Valentao P., Andrade P.B. (2013). Ellagic acid and derivatives from *Cochlospermum angolensis* Welw. Extracts: HPLC-DAD-ESI/MS^n^ profiling, quantification and in vitro anti-depressant, anti-cholinesterase and anti-oxidant activities. Phytochem. Anal..

[30] Bedel H.A., Kencebay Manas C., Ozbey G., Usta C. (2018). The antidepressant-like activity of ellagic acid and its effect on hippocampal brain derived neurotrophic factor levels in mouse depression models. Nat. Prod. Res..

[31] Dhingra D., Chhillar R. (2012). Antidepressant-like activity of ellagic acid in unstressed and acute immobilization-induced stressed mice. Pharmacol. Rep..

[32] Dhingra D., Jangra A. (2014). Antiepileptic activity of ellagic acid, a naturally occurring polyphenolic compound, in mice. J. Funct. Foods.

[33] Gonzalez-Barrio R., Truchado P., Ito H., Espin J.C., Tomas-Barberan F.A. (2011). UV and MS identification of Urolithins and Nasutins, the bioavailable metabolites of ellagitannins and ellagic acid in different mammals. J. Agric. Food Chem..

[34] Seeram N.P., Lee R., Heber D. (2004). Bioavailability of ellagic acid in human plasma after consumption of ellagitannins from pomegranate (*Punica granatum* L.) juice. Clin. Chim. Acta.

[35] Tomas-Barberan F.A., Gonzalez-Sarrias A., Garcia-Villalba R., Nunez-Sanchez M.A., Selma M.V., Garcia-Conesa M.T., Espin J.C. (2017). Urolithins, the rescue of “old” metabolites to understand a “new” concept: Metabotypes as a nexus among phenolic metabolism, microbiota dysbiosis, and host health status. Mol. Nutr. Food Res..

[36] Espin J.C., Gonzalez-Barrio R., Cerda B., Lopez-Bote C., Rey A.I., Tomas-Barberan F.A. (2007). Iberian pig as a model to clarify obscure points in the bioavailability and metabolism of ellagitannins in humans. J. Agric. Food Chem..

[37] Bala I., Bhardwaj V., Hariharan S., Kharade S.V., Roy N., Ravi Kumar M.N. (2006). Sustained release nanoparticulate formulation containing antioxidant-ellagic acid as potential prophylaxis system for oral administration. J. Drug Target..

[38] El-Missiry M.A., Othman A.I., Amer M.A., Sedki M., Ali S.M., El-Sherbiny I.M. (2020). Nanoformulated ellagic acid ameliorates pentylenetetrazol-induced experimental epileptic seizures by modulating oxidative stress, inflammatory cytokines and apoptosis in the brains of male mice. Metab. Brain Dis..

[39] Yuan T., Ma H., Liu W., Niesen D.B., Shah N., Crews R., Rose K.N., Vattem D.A., Seeram N.P. (2016). Pomegranate’s Neuroprotective Effects against Alzheimer’s Disease Are Mediated by Urolithins, Its Ellagitannin-Gut Microbial Derived Metabolites. ACS Chem. Neurosci..

[40] Gasperotti M., Passamonti S., Tramer F., Masuero D., Guella G., Mattivi F., Vrhovsek U. (2015). Fate of microbial metabolites of dietary polyphenols in rats: Is the brain their target destination?. ACS Chem. Neurosci..

[41] Yan L., Yin P., Ma C., Liu Y. (2014). Method development and validation for pharmacokinetic and tissue distributions of ellagic acid using ultrahigh performance liquid chromatography-tandem mass spectrometry (UPLC-MS/MS). Molecules.

[42] Espin J.C., Larrosa M., Garcia-Conesa M.T., Tomas-Barberan F. (2013). Biological significance of urolithins, the gut microbial ellagic Acid-derived metabolites: The evidence so far. Evid Based Complement. Alternat. Med..

[43] Lei F., Xing D.M., Xiang L., Zhao Y.N., Wang W., Zhang L.J., Du L.J. (2003). Pharmacokinetic study of ellagic acid in rat after oral administration of pomegranate leaf extract. J. Chromatogr. B Analyt. Technol. Biomed. Life Sci..

[44] Smart R.C., Huang M.T., Chang R.L., Sayer J.M., Jerina D.M., Conney A.H. (1986). Disposition of the naturally occurring antimutagenic plant phenol, ellagic acid, and its synthetic derivatives, 3-*O*-decylellagic acid and 3,3’-di-*O*-methylellagic acid in mice. Carcinogenesis.

[45] Teel R.W., Martin R.M. (1988). Disposition of the plant phenol ellagic acid in the mouse following oral administration by gavage. Xenobiotica.

[46] Press R.E., Hardcastle D. (1969). Some physico-chemical properties of ellagic acid. J. Appl. Chem..

[47] Teel R.W. (1987). Distribution and metabolism of ellagic acid in the mouse following intraperitoneal administration. Cancer Lett..

[48] White H.S. (1998). Chemoconvulsants. Neuropharmacology Methods in Epilepsy Research.

[49] Löscher W. (2009). Preclinical assessment of proconvulsant drug activity and its relevance for predicting adverse events in humans. Eur. J. Pharmacol..

[50] Castel-Branco M.M., Alves G.L., Figueiredo I.V., Falcao A.C., Caramona M.M. (2009). The maximal electroshock seizure (MES) model in the preclinical assessment of potential new antiepileptic drugs. Methods Find. Exp. Clin. Pharmacol..

[51] Li G., Bauer S., Nowak M., Norwood B., Tackenberg B., Rosenow F., Knake S., Oertel W.H., Hamer H.M. (2011). Cytokines and epilepsy. Seizure.

[52] Sudha K., Rao A.V., Rao A. (2001). Oxidative stress and antioxidants in epilepsy. Clin. Chim. Acta.

[53] Yang N., Guan Q.W., Chen F.H., Xia Q.X., Yin X.X., Zhou H.H., Mao X.Y. (2020). Antioxidants Targeting Mitochondrial Oxidative Stress: Promising Neuroprotectants for Epilepsy. Oxid Med. Cell Longev..

[54] Vishnoi S., Raisuddin S., Parvez S. (2016). Glutamate Excitotoxicity and Oxidative Stress in Epilepsy: Modulatory Role of Melatonin. J. Environ. Pathol. Toxicol. Oncol..

[55] Mukhopadhyay B., Gavel R., Gongopadhyay A.N., Vashistha P., Rani A., Mishra S.P. (2016). Correlation of Oxidative Damage with Pro-Inflammatory Markers (IL-6, TNF-α) in Meningocele. J. Clin. Diagn. Res..

[56] Pauletti A., Terrone G., Shekh-Ahmad T., Salamone A., Ravizza T., Rizzi M., Pastore A., Pascente R., Liang L.P., Villa B.R. (2017). Targeting oxidative stress improves disease outcomes in a rat model of acquired epilepsy. Brain.

[57] Patsoukis N., Zervoudakis G., Panagopoulos N.T., Georgiou C.D., Angelatou F., Matsokis N.A. (2004). Thiol redox state (TRS) and oxidative stress in the mouse hippocampus after pentylenetetrazol-induced epileptic seizure. Neurosci. Lett..

[58] Han D.H., Lee M.J., Kim J.H. (2006). Antioxidant and apoptosis-inducing activities of ellagic acid. Anticancer Res..

[59] Kiasalari Z., Heydarifard R., Khalili M., Afshin-Majd S., Baluchnejadmojarad T., Zahedi E., Sanaierad A., Roghani M. (2017). Ellagic acid ameliorates learning and memory deficits in a rat model of Alzheimer’s disease: An exploration of underlying mechanisms. Psychopharmacology.

[60] Farbood Y., Sarkaki A., Dolatshahi M., Taqhi Mansouri S.M., Khodadadi A. (2015). Ellagic Acid Protects the Brain Against 6-Hydroxydopamine Induced Neuroinflammation in a Rat Model of Parkinson’s Disease. Basic Clin. Neurosci..

[61] Taghi Mansouri M., Naghizadeh B., Ghorbanzadeh B., Farbood Y. (2013). Central and peripheral antinociceptive effects of ellagic acid in different animal models of pain. Eur. J. Pharmacol..

[62] Ghorbanzadeh B., Mansouri M.T., Hemmati A.A., Naghizadeh B., Mard S.A., Rezaie A. (2014). Involvement of l-arginine/NO/cGMP/K(ATP) channel pathway in the peripheral antinociceptive actions of ellagic acid in the rat formalin test. Pharmacol. Biochem. Behav..

[63] Mansouri M.T., Naghizadeh B., Ghorbanzadeh B. (2014). Involvement of opioid receptors in the systemic and peripheral antinociceptive actions of ellagic acid in the rat formalin test. Pharmacol. Biochem. Behav..

[64] Olgar Y., Ozturk N., Usta C., Puddu P.E., Ozdemir S. (2014). Ellagic acid reduces L-type Ca2+ current and contractility through modulation of NO-GC-cGMP pathways in rat ventricular myocytes. J. Cardiovasc. Pharmacol..

[65] Rundfeldt C., Koch R., Richter A., Mevissen M., Gerecke U., Löscher W. (1995). Dose-dependent anticonvulsant and proconvulsant effects of nitric oxide synthase inhibitors on seizure threshold in a cortical stimulation model in rats. Eur. J. Pharmacol..

[66] Fedele E., Raiteri M. (1999). In vivo studies of the cerebral glutamate receptor/NO/cGMP pathway. Prog. Neurobiol..

[67] Banach M., Piskorska B., Czuczwar S.J., Borowicz K.K. (2011). Nitric oxide, epileptic seizures, and action of antiepileptic drugs. CNS Neurol. Disord. Drug Targets.

[68] Prast H., Philippu A. (2001). Nitric oxide as modulator of neuronal function. Prog. Neurobiol..

[69] Karim N., Khan I., Khan H., Ayub B., Abdel-Halim H., Gavande N. (2018). Anxiolytic Potential of Natural Flavonoids. SM J. Endocrinol. Metab..

[70] Svob Strac D., Pivac N., Smolders I.J., Fogel W.A., De Deurwaerdere P., Di Giovanni G. (2016). Monoaminergic Mechanisms in Epilepsy May Offer Innovative Therapeutic Opportunity for Monoaminergic Multi-Target Drugs. Front. Neurosci..

[71] Binder D.K. (2004). The role of BDNF in epilepsy and other diseases of the mature nervous system. Adv. Exp. Med. Biol..

[72] Bhandary B.S.K., Sharmila K.P., Kumari N.S., Bhat S.V. (2013). Acute and subacute toxicity study of the ethanol extracts of *Punica granatum* (linn). Whole fruit and seeds and synthetic ellagic acid in swiss albino mice. Asian J. Pharm. Clin. Res..

[73] Patel C., Dadhaniya P., Hingorani L., Soni M.G. (2008). Safety assessment of pomegranate fruit extract: Acute and subchronic toxicity studies. Food Chem. Toxicol..

[74] Socała K., Wlaź P., Vohora D. (2021). Acute Seizure Tests Used in Epilepsy Research: Step-by-Step Protocol of the Maximal Electroshock Seizure (MES) Test, the Maximal Electroshock Seizure Threshold (MEST) Test, and the Pentylenetetrazole (PTZ)-Induced Seizure Test in Rodents. Experimental and Translational Methods to Screen Drugs Effective Against Seizures and Epilepsy.

[75] Kimball A.W., Burnett W.T., Doherty D.G. (1957). Chemical protection against ionizing radiation. I. Sampling methods for screening compounds in radiation protection studies with mice. Radiat. Res..

[76] Boissier J.R., Tardy J., Diverres J.C. (1960). Une nouvelle methode simple pour explorer l’action ‘tranquilistante’: Le test de la cheminee. Med. Exp..

